# Pain

**Published:** 1915-06

**Authors:** William I. French, Reginald L. Felton


### PAIN.

READ BEFORE THE STUDENTS' DENTAL SOCIETY OF THE UNIVERSITY OF MICHIGAN, APRIL 5, 1915.

BY WILLIAM I. FRENCH AND REGINALD L. FELTON.

No other phenomena connected with the life history of the human body has been so great a factor in the historic development of medicine and dentistry as pain. We must admit that we know less about the nervous system than about the other systems of the human body, and the functions of the brain constitute almost as great a mystery today as it was to our medical forefathers.

It is very difficult to give a satisfactory definition of pain. It may, however, be regarded as a peculiar discomfort or suffering, caused by an abnormal disturbance of the sensory nerves or nerve cells, which induces a condition of over stimulation or irritation. Thus any of our sensations may become painful, if the stimulus is sufficiently strong or prolonged. From a restricted philosophical point of view, pain may be considered as a reaction of the organism in part, or in whole, to harmful influences. We may have pain in consciousness connected with its more complex process, such as fear, anxiety, or anger. With the mind intently fixed on the idea that pain is to be inflicted, the suffering is always more acute. The age of the patient usually modifies the susceptibility to pain; old patients being less susceptible than the young, owing to the sluggish condition of the nervous system. Pain susceptibility is also markedly influenced by the social condition; such as refinement, educational status, and occupation. A highly refined individual should be more susceptible than a laborer or farm hand, owing to a more highly developed and sensitive nervous system of the former, except that ignorant persons suffer more from fear than more intellectual people.

Prevention of pain has been sought for by all people, and in all eras. Way back in the early ages, history records that the ancients knew of the use of opium and other narcotics, but unfortunately nothing was really done until about fifty years ago. At that time local anaesthesia began to receive its due recognition. However, Horace Well did some work with nitrous oxide about 1845, and to him must be given the credit of being the first person to use nitrous oxide as a general anaesthetic.

It is true that for ages, people have been attempting to find a method by which pain could be eliminated. We are certain that pain has many disadvantages, and that more progress could be made from an operative standpoint if pain could be safely abolished. But has pain no advantages? I would say that it has. First, let us take up its advantages. Perhaps the most important advantage of pain to the dentist is that it brings the patient to the dental office. If for no other reason, a person will come to a dentist's office, it is to be relieved of pain. As has been stated before, this is the most important advantage of pain, because if he is once under the dentist's observation, then advice and unlimited services may be rendered. These services may be accelerated by the diagnostic symptoms obtained from pain. The source of the trouble may be located and diagnosed by means of thermal and electrical changes. Also, the presence of a throbbing pain locates the point of hyperemia. The presence and kind of abscesses may be diagnosed by pain. An alveolar abscess, if neglected, would follow its destructive course, involving its surrounding tissues, and eventually lead to general systemic trouble.

It is true that pain has its advantages, but it also has numerous disadvantages. To the average person, the dental office is much dreaded, because of the pain associated with the dental chair. Rather than to be subjected to pain, people neglect their teeth, giving the progress of caries and other dental diseases, an opportunity to go on unhindred, until they have reached a point beyond repair. Not only is it a disadvantage to the patient, but also to the operator. He is hindered in his operations, not being able to do his work thoroughly. If it were not for pain, much better work could be produced with much more lasting effects. Dental fatigue and shock are brought on by pain and thoughts of pain. Shock is dependent upon too sudden and too painful stimulation of the afferent nerves. Dental fatigue and dental shock differ only in degree. Dental fatigue bordering on shock, must be handled with the greatest of care by the dentist, to avert shock. From a financial standpoint, pain is a great loss to the patient, in that they dread coming to the dental office, allowing caries and dental trouble to develop to such extensive areas, that the restoration is much greater than if they had come at an early date, while the conditions were in a developmental stage. The cost of restoring the lost tissues being much less in the developmental, than in the advanced stage. Pain not only produces a financial loss to the patient, but also to the operator. Many people would rather stay away from the dental office, allowing their teeth to decay until only extraction is the last resort. Also the operator is obliged to spend more time in performing his operations and time is a valuable asset to the dental practitioner.

The foregoing discussion shows that pain has indispensable advantages, as well as many disadvantages. It is with the disadvantages in mind, that the extensive work in local and general anaesthesia is being carried on. The general surgeon employes anaesthetics to prevent shock. The dental surgeon should employ anaesthetics to lessen pain, prevent fatigue, and avert shock. With these facts in mind, the use of nitrous oxide, somnoform, novocaine, conductive anaesthesia, desensitizing paste, and the like, are employed in the modern dental office to a very great advantage. They not only make it possible for the practitioner to carry on extensive operations, and to do them in a shorter time, but they have made it possible, if all the methods of anaesthesia are taken advantage of, to prevent pain during dental operations, and this is a service to humanity, the value of which can not be estimated.

